# Optimal direct oral anticoagulant for upper gastrointestinal endoscopic submucosal dissection

**DOI:** 10.1007/s00535-024-02171-2

**Published:** 2024-11-27

**Authors:** Yoshitaka Ono, Waku Hatta, Kunio Tarasawa, Yohei Ogata, Hiroko Abe, Isao Sato, Yutaka Hatayama, Masahiro Saito, Xiaoyi Jin, Kaname Uno, Tomoyuki Koike, Akira Imatani, Shin Hamada, Kenji Fujimori, Kiyohide Fushimi, Atsushi Masamune

**Affiliations:** 1 Division of Gastroenterology, Tohoku University Graduate School of Medicine, 1-1 Seiryo-Machi, Aoba-Ku, Sendai, 980-8574 Japan; 2 Department of Health Administration and Policy, Tohoku University Graduate School of Medicine, 2-1 Seiryo-Machi, Aoba-Ku, Sendai, 980-8575 Japan; 3 Department of Health Policy and Informatics, Graduate School of Medical and Dental Sciences, Tokyo Medical and Dental University, S1560/S1568 M&D Tower 1-5-45 Yushima, Bunkyo-Ku, Tokyo 113-8519 Japan

**Keywords:** Direct oral anticoagulant, Endoscopic submucosal dissection, Delayed bleeding, Ischemic stroke, Rivaroxaban

## Abstract

**Background:**

The patients taking direct oral anticoagulants (DOACs) are at high risk for developing ischemic stroke and delayed bleeding in upper gastrointestinal endoscopic submucosal dissection (ESD). We aimed to identify the optimal DOAC based on both adverse events in upper gastrointestinal ESD.

**Methods:**

A retrospective population-based cohort study was conducted using the Diagnosis Procedure Combination database in Japan. We included patients on a DOAC undergoing upper gastrointestinal ESD between 2012 and 2021. The primary outcomes were ischemic stroke occurring after upper gastrointestinal ESD and delayed bleeding in gastroduodenal and esophageal ESD. Inverse probability weightings were applied to balance the four DOAC groups (dabigatran, rivaroxaban, apixaban, and edoxaban), and logistic regression analyses were performed to compare the outcomes.

**Results:**

We analyzed 9729 patients on a DOAC undergoing upper gastrointestinal ESD. Ischemic stroke developed after upper gastrointestinal ESD in 1.4%, 0.7%, 0.6%, and 0.8% of patients taking dabigatran, rivaroxaban, apixaban, and edoxaban, respectively, after weighting. Rivaroxaban and apixaban showed significantly lower risk of ischemic stroke compared with dabigatran (odds ratio, 0.15 and 0.12, respectively) in standard doses. The delayed bleeding developed after gastroduodenal ESD in 7.6%, 14.6%, 19.2%, and 17.3% of patients taking each DOAC, respectively, with the lowest risk in dabigatran, followed by rivaroxaban. A similar pattern was observed in delayed bleeding in esophageal ESD (3.2%, 5.4%, 7.5%, and 5.5% in each DOAC), but with no significant results.

**Conclusions:**

Rivaroxaban might be an optimal DOAC for upper gastrointestinal ESD showing a lower risk for both ischemic stroke and delayed bleeding.

**Supplementary Information:**

The online version contains supplementary material available at 10.1007/s00535-024-02171-2.

## Introduction

Endoscopic submucosal dissection (ESD) is a widely used minimally invasive procedure for treating early-stage gastrointestinal (GI) tumors in the Eastern Asian countries [1, 2], with favorable long-term outcomes [3, 4]. This technique is also gaining recognition in Western countries [5]. One of the major adverse events in upper GI ESD is delayed bleeding, and the overall rates of this adverse event are 0.3–1.3% [6–8], 5.1% [9], and 3.4–6.3% [10–12] in esophageal, gastric, and duodenal ESD, respectively. Patients at high risk for thromboembolism are recommended to take anticoagulants and these agents generally pose the highest risk for delayed bleeding in gastric ESD [13, 14]. Most thromboembolisms include ischemic stroke in gastric ESD [15].

Among anticoagulants, direct oral anticoagulant (DOAC) prescription has dramatically increased over the past decade [16]. For example, a study showed that DOACs were prescribed for about 86% of patients (4128/4790) with acute symptomatic venous thromboembolism between 2015 and 2020 [17]. Accordingly, research has focused on identifying safe and effective DOACs in some fields [18–20]. However, there is no clear consensus on optimal DOAC for upper GI ESD. While some studies evaluated DOACs in this context, a nationwide multicenter retrospective study in Japan investigating delayed bleeding rates of four DOACs in gastric ESD [21] lacked sufficient cases for direct comparison. Additionally, a previous report on esophageal ESD with a small sample of DOAC users (16 cases) also made comparing the four DOACs difficult, though delayed bleeding was notably high at 16% [8]. Despite guidelines emphasizing the risk of thromboembolism over bleeding due to its seriousness [22], no studies evaluated the risk of thromboembolism or ischemic stroke among the four DOACs in upper GI ESD because of limited case numbers.

The Diagnosis Procedure Combination (DPC) database used in our study group includes administrative claims and admission and discharge abstracts obtained from over 1000 participating hospitals throughout Japan, covering approximately 90% of tertiary hospitals and 50% of acute-care hospitalizations (about 7 million annually) [23, 24]. Thus, this database may allow the comparison of the risks of ischemic stroke and delayed bleeding among the four DOACs in upper GI ESD. The present study aimed to identify the optimal DOAC for upper GI ESD using the DPC database.

## Methods

### Study design and database

This retrospective cohort study used the DPC database that includes data on patient demographics, diagnoses, comorbidities upon admission and complications during hospitalization that were coded with the International Classification of Diseases and Related Health Problems, 10th Revision codes [25] and supplemented by text data in Japanese; procedures that were coded with the Medical Intervention Classification master code [26] (treatment code), medications, including drugs that were administered daily, and unique hospital identifier. This study was approved by the Ethics Committee of Tohoku University Graduate School of Medicine and adheres to the Declaration of Helsinki. Informed consent was waived due to the anonymity of the data.

### Study population

**Fig. 1:**
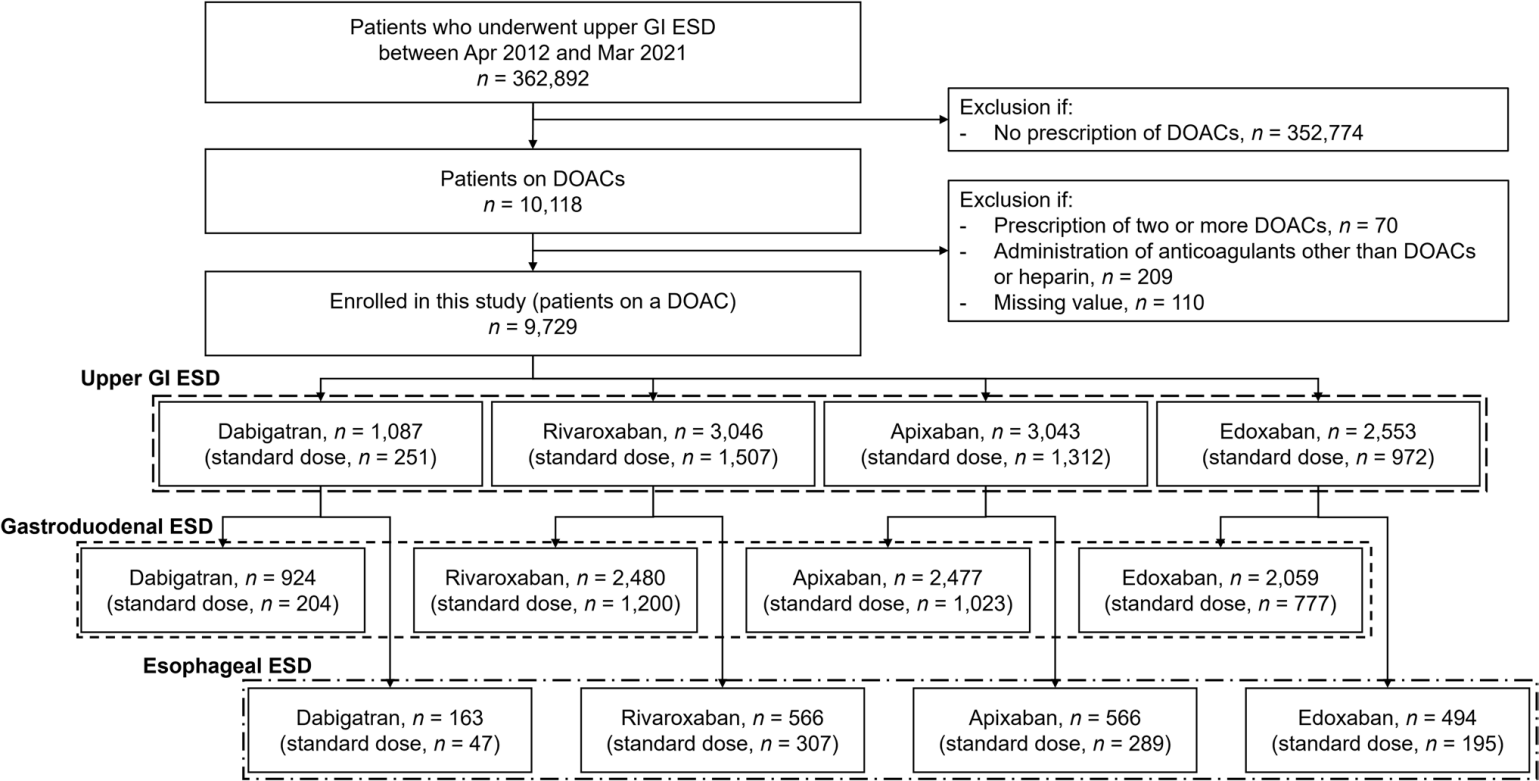
Flow diagram of patient enrollment. *GI* gastrointestinal, *ESD* endoscopic submucosal dissection, *DOAC* direct oral anticoagulant

We initially extracted data of patients who experienced upper GI ESD once during hospitalization and were correspondingly prescribed DOACs between April 2012 and March 2021. The patients on warfarin were not included in the study due to the significant decline in its prescription over the past decade [16, 17]. This study permitted multiple entries for the same patients. The exclusion criteria were 1) patients prescribed two or more DOACs; 2) those receiving anticoagulants other than DOACs or heparin; and 3) those with missing data (Fig. 1).

### Doses of DOACs

The standard daily doses in Japan for dabigatran, rivaroxaban, apixaban, and edoxaban are 300 mg, 15 mg, 10 mg, and 60 mg, respectively. Supplementary Table 1 outlines the characteristics and dosage reduction criteria for these four DOACs in Japan.

### Data collection and variables

We collected data on gastroduodenal and esophageal ESD, delayed bleeding, ischemic stroke, age, sex, body mass index (BMI), comorbidities for Charlson comorbidity index (CCI) [27] and CHADS2 score [28], annual hospital volumes, and concurrent medication that may be associated with delayed bleeding and ischemic stroke. The DPC database is highly specific and sensitive regarding the procedure records and is highly specific but moderately sensitive for most diagnoses [29].

Concurrent medications included proton pump inhibitors (PPIs), vonoprazan, antiplatelet agents (aspirin, P2Y12 receptor antagonist [P2Y12RA], cilostazol, and other antiplatelet agents), DOACs (dabigatran, rivaroxaban, apixaban, and edoxaban), heparin, nonsteroidal anti-inflammatory drugs (NSAIDs), corticosteroids, and mucosal protective agents.

### Outcomes of interest

This study focused on ischemic stroke and delayed bleeding in upper GI ESD. The delayed bleeding in upper GI ESD was classified into two categories: gastroduodenal and esophageal ESD, as these procedures are known to have different rates of delayed bleeding [6–9]. Consequently, this study evaluated three outcomes: ischemic stroke in upper GI ESD, delayed bleeding in gastroduodenal ESD, and delayed bleeding in esophageal ESD. Delayed bleeding was defined as bleeding that requires endoscopic hemostasis and/or blood transfusion between 2 and 30 days post-treatment, based on prior research [30–32]. Ischemic stroke was defined by its specific coding with an observation period extending up to 30 days after ESD, based on the previous study [33]. These outcomes were evaluated against four DOACs, regardless of dosage.

### Statistical analysis

Continuous variables were presented as means with standard deviations and categorical variables were presented as counts with proportions.

We employed the inverse probability weighting (IPW) method to achieve an unbiased comparison across four DOACs, which is especially suitable in cases with numerous treatment alternatives [34, 35]. We utilized generalized boosted models, which employ an iterative process with 10,000 regression trees, to calculate weights for best balance among the treatment groups [36]. These weights were developed to generate estimates that represent the average treatment effects of the groups. The covariates in the propensity model included age, sex, BMI, CCI, annual hospital volume, PPIs, vonoprazan, heparin, aspirin, P2Y12RA, cilostazol, other antiplatelet agents, NSAIDs, corticosteroids, and mucosal protective agents. Chronic kidney disease with hemodialysis demonstrated a high risk for delayed bleeding in gastric ESD [14], but it was excluded from the covariates because DOACs are not prescribed to patients with chronic kidney disease on hemodialysis in Japan. Standardized mean differences (SMDs) were calculated to assess the balance of patient characteristics among the four groups, with SMDs of ≤ 0.10 considered indicative of good balances and ≤ 0.20 as acceptable [37]. After applying IPW, we assessed the risk of ischemic stroke or delayed bleeding among the four DOAC groups with logistic regression analysis. This study considered a two-sided *p*-value of < 0.05 indicate statistical significance. The *p*-values were presented along with the effect of correction utilizing the Holm’s method, which is a step-down modified Bonferroni’s correction method, in multiple comparisons. The significance level after Holm’s correction is indicated in each figure legend when the *p*-value is < 0.05. All statistical analyses were carried out using R version 3.6.1 for Windows (The R Foundation for Statistical Computing, Vienna, Austria).

### Sensitivity analyses

We conducted two sensitivity analyses to confirm the robustness of the acquired results. The primary analysis included both the standard and reduced doses of DOACs. Given that the dosage reduction criteria differ among the four DOACs, variations in the rates of standard and reduced doses may influence the results. Hence, we evaluated the risk of ischemic stroke in upper GI ESD and that of delayed bleeding in gastroduodenal ESD among the four DOACs restricted to the standard dose. We did not evaluate delayed bleeding in esophageal ESD because of the small number of cases. Additionally, since heparin bridging may impact the incidence of ischemic stroke and delayed bleeding in patients on DOACs, we compared the risks of ischemic stroke in upper GI ESD and delayed bleeding in gastroduodenal ESD and esophageal ESD were compared among the four DOACs in patients who did not use heparin.

## Results

### Patient characteristics

Figure 1 illustrates the flow diagram of patient enrollment, with a total of 9729 patients analyzed in this study. Table 1 details the patient characteristics prior to weighting. Three IPWs for patients with upper GI ESD, those with gastroduodenal ESD, and those with esophageal ESD were conducted to balance the four DOAC groups for evaluating three outcomes. After applying IPW, all patient characteristics were well balanced with a maximum SMD of 0.070 (Table 1). The mean (standard deviation) length of hospitalization was 12.1 (14.4) days. The use of heparin decreased significantly after 2018 compared with the period before 2017 (8.9% vs. 27.9%, *p* < 0.001).

**Table 1:** Participant characteristics according to DOAC administration

	Dabigatran	Rivaroxaban	Apixaban	Edoxaban	SMD	
					Before IPW	After IPW
Upper GI ESD	*n* = 1,087	*n* = 3,046	*n* = 3,043	*n* = 2,553		
Age (y), mean (SD)	75.8 (6.8)	76.3 (6.9)	77.7 (6.8)	77.0 (7.3)	0.154	0.021
*Sex*, *n* (%)						
Male	871 (89.8)	2,122 (83.4)	2,062 (81.8)	1,620 (78.1)	0.168	0.036
Female	216 (10.2)	924 (16.6)	981 (18.2)	933 (21.9)		
BMI (kg/m^2^), mean (SD)	23.7 (3.3)	23.7 (3.4)	23.5 (4.0)	23.3 (3.6)	0.069	0.016
CCI, mean (SD)	1.0 (1.1)	1.0 (1.1)	1.2 (1.2)	1.1 (1.2)	0.099	0.028
Hospital volume, mean (SD)	72.8 (58.9)	67.5 (54.7)	66.3 (56.5)	64.7 (51.4)	0.076	0.004
*Drug use*, *n* (%)						
Heparin	215 (19.8)	446 (14.6)	549 (18.0)	353 (13.8)	0.095	0.026
Aspirin	97 (8.9)	315 (10.3)	325 (10.7)	269 (10.5)	0.031	0.018
P2Y12RA	39 (3.6)	127 (4.2)	172 (5.7)	94 (3.7)	0.053	0.018
Cilostazol	19 (1.7)	63 (2.1)	78 (2.6)	78 (3.1)	0.048	0.016
Other antiplatelet drugs	39 (3.6)	91 (3.0)	99 (3.3)	80 (3.1)	0.018	0.004
PPIs	963 (88.6)	2,609 (85.7)	2,595 (85.3)	2,119 (83.0)	0.082	0.020
Vonoprazan	476 (43.8)	1,566 (51.4)	1,690 (55.5)	1,585 (62.1)	0.199	0.027
Mucosal protective agents	749 (68.9)	2,092 (68.7)	2,133 (70.1)	1,737 (68.0)	0.023	0.025
NSAIDs	80 (7.4)	212 (7.0)	204 (6.7)	197 (7.7)	0.022	0.017
Corticosteroids	59 (5.4)	174 (5.7)	189 (6.2)	230 (9.0)	0.073	0.013
CHADS2 score	1.4 (1.1)	1.4 (1.0)	1.6 (1.1)	1.5 (1.1)	0.114	0.037
Gastroduodenal ESD	*n* = 924	*n* = 2,480	*n* = 2,477	*n* = 2,059		
Age (y), mean (SD)	76.3 (6.7)	76.7 (6.8)	78.2 (6.6)	77.6 (7.1)	0.162	0.031
*Sex*, *n* (%)					0.163	0.050
Male	826 (89.4)	2,062 (83.1)	2,021 (81.6)	1,606 (78.0)		
Female	98 (10.6)	418 (16.9)	456 (18.4)	453 (22.0)		
BMI (kg/m^2^), mean (SD)	23.8 (3.4)	23.9 (3.5)	23.6 (4.1)	23.5 (3.6)	0.054	0.014
CCI, mean (SD)	0.9 (1.0)	1.0 (1.1)	1.2 (1.3)	1.1 (1.2)	0.111	0.042
Hospital volume, mean (SD)	79.9 (59.9)	75.5 (55.8)	74.4 (58.2)	72.9 (52.3)	0.065	0.005
*Drug use*, *n* (%)						
Heparin	179 (19.4)	352 (14.2)	444 (17.9)	284 (13.8)	0.092	0.027
Aspirin	83 (9.0)	268 (10.8)	279 (11.3)	230 (11.2)	0.040	0.034
P2Y12RA	32 (3.5)	105 (4.2)	149 (6.0)	72 (3.5)	0.067	0.024
Cilostazol	17 (1.8)	56 (2.3)	67 (2.7)	62 (3.0)	0.043	0.023
Other antiplatelet drugs	36 (3.9)	70 (2.8)	78 (3.1)	65 (3.2)	0.030	0.007
PPIs	822 (89.0)	2,128 (85.8)	2,113 (85.3)	1,710 (83.1)	0.088	0.033
Vonoprazan	427 (46.2)	1,363 (55.0)	1,490 (60.2)	1,359 (66.0)	0.220	0.031
Mucosal protective agents	646 (69.9)	1,719 (69.3)	1,785 (72.1)	1,430 (69.5)	0.032	0.019
NSAIDs	54 (5.8)	147 (5.9)	145 (5.9)	149 (7.2)	0.029	0.014
Corticosteroids	42 (4.5)	110 (4.4)	130 (5.2)	161 (7.8)	0.076	0.023
CHADS2 score	1.4 (1.1)	1.5 (1.0)	1.7 (1.1)	1.5 (1.1)	0.122	0.038
Esophageal ESD)	*n* = 163	*n* = 566	*n* = 566	*n* = 494		
Age (y), mean (SD)	73.0 (6.7)	74.2 (7.0)	75.5 (6.9)	74.2 (7.4)	0.178	0.035
*Sex*, *n* (%)					0.099	0.068
Male	154 (94.5)	531 (93.8)	517 (91.3)	445 (90.1)		
Female	9 (5.5)	35 (6.2)	49 (8.7)	49 (9.9)		
BMI (kg/m^2^), mean (SD)	23.2 (3.2)	23.0 (3.3)	22.8 (3.3)	22.4 (3.3)	0.140	0.042
CCI, mean (SD)	1.1 (1.2)	1.0 (1.2)	1.1 (1.2)	1.2 (1.3)	0.063	0.029
Hospital volume, mean (SD)	32.3 (29.8)	32.5 (30.4)	30.7 (28.4)	30.8 (28.8)	0.041	0.034
*Drug use*, *n* (%)						
Heparin	36 (22.1)	94 (16.6)	105 (18.6)	69 (14.0)	0.115	0.040
Aspirin	14 (8.6)	47 (8.3)	46 (8.1)	39 (7.9)	0.014	0.009
P2Y12RA	7 (4.3)	22 (3.9)	23 (4.1)	22 (4.5)	0.016	0.022
Cilostazol	2 (1.2)	7 (1.2)	11 (1.9)	16 (3.2)	0.078	0.042
Other antiplatelet drugs	3 (1.8)	21 (3.7)	21 (3.7)	15 (3.0)	0.063	0.031
PPIs	141 (86.5)	481 (85.0)	482 (85.2)	409 (82.8)	0.052	0.040
Vonoprazan	49 (30.1)	203 (35.9)	200 (35.3)	226 (45.7)	0.165	0.054
Mucosal protective agents	103 (63.2)	373 (65.9)	348 (61.5)	307 (62.1)	0.050	0.070
NSAIDs	26 (16.0)	65 (11.5)	59 (10.4)	48 (9.7)	0.099	0.030
Corticosteroids	17 (10.4)	64 (11.3)	59 (10.4)	69 (14.0)	0.059	0.033
CHADS2 score	1.2 (1.1)	1.2 (1.0)	1.4 (1.1)	1.3 (1.1)	0.119	0.053

*DOACs* direct oral anticoagulants, *SMD* standardized mean difference, *IPW* inverse probability weighting, *ESD* endoscopic submucosal dissection, *SD* standard deviation, *BMI* body mass index, *CCI* Charlson comorbidity index, *P2Y12RA* P2Y12 receptor antagonist, *PPIs* proton pump inhibitors, *NSAIDs* nonsteroidal anti-inflammatory drugs

### Ischemic stroke in upper GI ESD

**Fig. 2:**
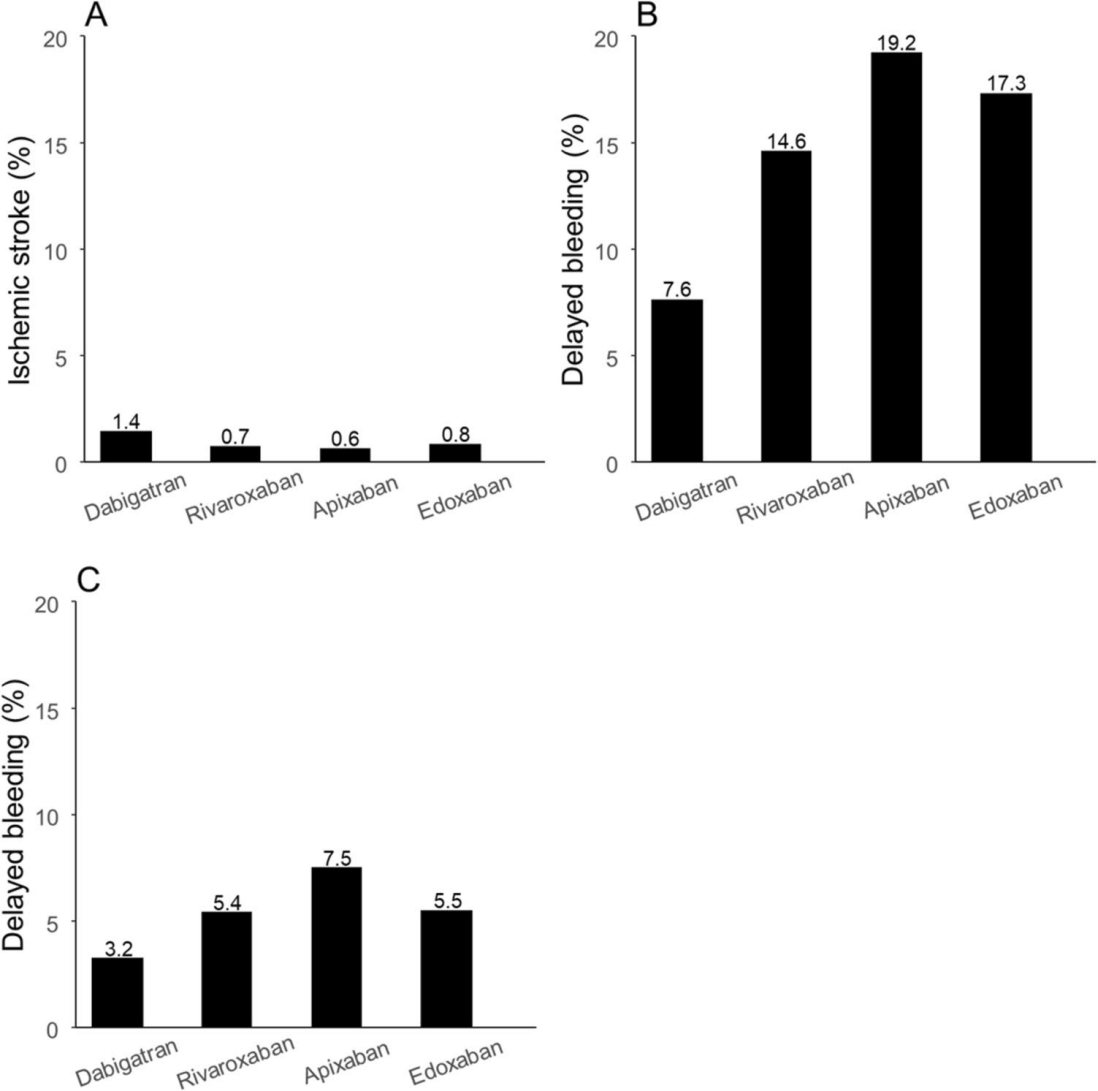
Rates of ischemic stroke in upper GI ESD and delayed bleeding in gastroduodenal and esophageal ESD in the four DOACs after IPW. **a** Rate of ischemic stroke in upper GI ESD. **b** Rate of delayed bleeding in gastroduodenal ESD. **c** Rate of delayed bleeding in esophageal ESD. *GI* gastrointestinal, *ESD* endoscopic submucosal dissection, *DOAC* direct oral anticoagulant, *IPW* inverse probability weighting

**Fig. 3:**
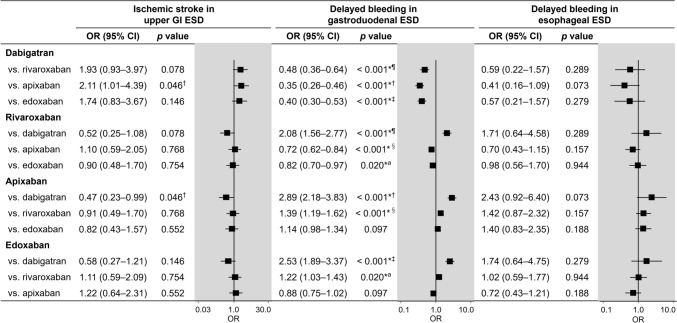
Risk of ischemic stroke in upper GI ESD and delayed bleeding in gastroduodenal and esophageal ESD among the four DOACs. *Statistically sigificant after Holm’s correction.†The significance level after Holm’s correction was set as *p* < 0.0083. ‡The significance level after Holm’s correction was set as *p* < 0.010. ¶The significance level after Holm’s correction was set as *p* < 0.013. §The significance level after Holm’s correction was set as *p* < 0.017. ^a^The significance level after Holm’s correction was set as *p* < 0.025. *GI* gastrointestinal, *ESD* endoscopic submucosal dissection, *DOAC* direct oral anticoagulant, *OR* odds ratio, *CI* confidence interval

Ischemic stroke occurred after upper GI ESD in 1.4%, 0.7%, 0.6%, and 0.8% of patients taking dabigatran, rivaroxaban, apixaban, and edoxaban, respectively (rates according to the data presented in Fig. 2A). The risk of ischemic stroke in rivaroxaban (odds ratio [OR], 0.52), apixaban (OR: 0.47), and edoxaban (OR, 0.58) appeared lower in comparison with dabigatran, but it did not reach the statistical significance after correction for multiple testing (Fig. 3). No significant results were found in the comparisons among rivaroxaban, apixaban, and edoxaban.

### Delayed bleeding in gastroduodenal ESD

Delayed bleeding occurred after gastroduodenal ESD in 7.6%, 14.6%, 19.2%, and 17.3% of patients taking dabigatran, rivaroxaban, apixaban, and edoxaban, respectively (rates according to the data presented in Fig. 2B). Figure 3 illustrates the bleeding risk associated with each DOAC compared with the others. The risk of delayed bleeding was the lowest in dabigatran when compared with rivaroxaban, apixaban, and edoxaban, with ORs of 0.48, 0.35, and 0.40, respectively. Additionally, the risk of delayed bleeding with rivaroxaban was significantly lower than that of apixaban (OR, 0.72) and edoxaban (OR, 0.82).

### Delayed bleeding in esophageal ESD

Delayed bleeding occurred after esophageal ESD in 3.2%, 5.4%, 7.5%, and 5.5% of patients taking dabigatran, rivaroxaban, apixaban, and edoxaban, respectively (rates according to the data presented in Fig. 2C). The risk of delayed bleeding with dabigatran (OR: 0.41) compared with apixaban appeared lower; however, this difference was not statistically significant (Fig. 3).

### Sensitivity analysis

**Fig. 4:**
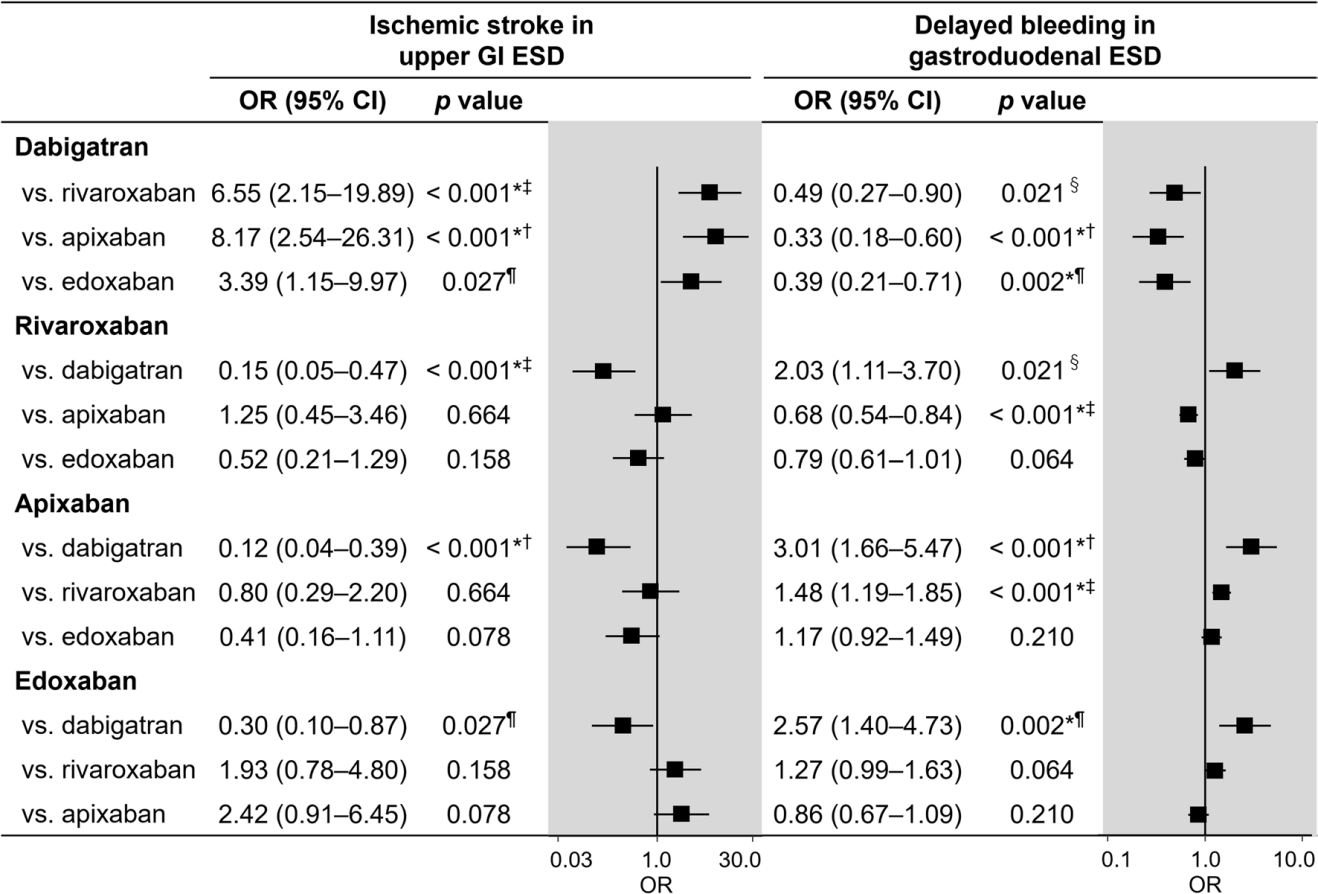
Risk of ischemic stroke in upper GI ESD and delayed bleeding in gastroduodenal ESD among the four DOACs limited to standard dose. *Statistically sigificant after Holm’s correction. †The significance level after Holm’s correction was set as *p* < 0.0083. ‡The significance level after Holm’s correction was set as *p* < 0.010. ¶The significance level after Holm’s correction was set as *p* < 0.013. §The significance level after Holm’s correction was set as *p* < 0.017. *GI* gastrointestinal, *ESD* endoscopic submucosal dissection, *DOAC* direct oral anticoagulant, *OR* odds ratio, *CI* confidence interval

In the analysis of DOACs limited to the standard dose, the balance of patient characteristics after weighting was either good or acceptable with a maximum SMD of 0.123 (Supplementary Table 2). Unlike the main analysis, the lower risk of ischemic stroke for upper GI ESD in rivaroxaban (OR, 0.15) and apixaban (OR, 0.12), compared with dabigatran, was statistically significant, whereas the result for edoxaban did not reach the statistical significance after correction for multiple testing (Fig. 4). The lower risk of delayed bleeding in gastroduodenal ESD for dabigatran, compared with apixaban (OR, 0.33) and edoxaban (OR, 0.39), as well as for rivaroxaban, compared with apixaban (OR, 0.68), remained significant (Fig. 4). Although the ORs for the risk of delayed bleeding for dabigatran, compared with rivaroxaban (0.49), and rivaroxaban, compared with edoxaban (0.79), were similar to those in the main analysis, these comparisons did not reach statistical significance after correction for multiple testing.

**Fig. 5:**
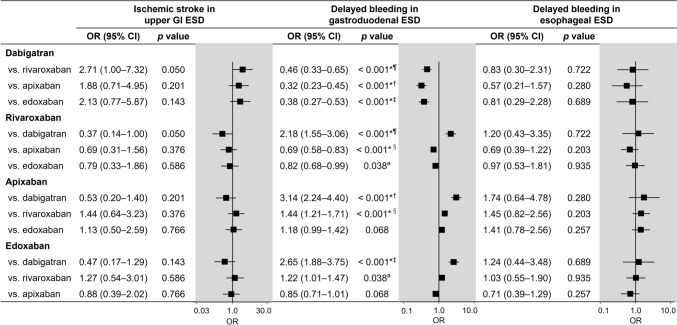
Risk of three clinical outcomes among DOACs when patients were limited to no use of heparin. *Statistically sigificant after Holm’s correction. †The significance level after Holm’s correction was set as *p* < 0.0083. ‡The significance level after Holm’s correction was set as *p* < 0.010. ¶The significance level after Holm’s correction was set as *p* < 0.013. §The significance level after Holm’s correction was set as *p* < 0.017. ^a^The significance level after Holm’s correction was set as *p* < 0.025. *DOAC* direct oral anticoagulant, *GI* gastrointestinal, *ESD* endoscopic submucosal dissection, *OR* odds ratio, *CI* confidence interval

The second sensitivity analysis compared three outcomes between any two of the four DOACs in patients not using heparin. The balance of patient characteristics after weighting was either good or acceptable with a maximum SMD of 0.110 (Supplementary Table 3). Most significant findings remained consistent with the main analysis; however, this sensitivity analysis did not reach the statistical significance after correction for multiple testing of outcome data in the comparison of rivaroxaban and edoxaban regarding the risk of delayed bleeding in gastroduodenal ESD, even though the OR (0.82) was the same as in the main analysis (Fig. 5).

## Discussion

The patients taking DOACs are at high risk for both ischemic stroke and delayed bleeding in upper GI ESD [8, 13, 14], making it essential to identify the optimal DOAC considering these adverse events. Evaluating the risk of ischemic stroke proved challenging due to the need for a very large number of cases. Thus, this study utilized the DPC database to address these issues.

This study includes two key clinical insights. First, dabigatran exhibited the lowest risk for delayed bleeding in gastroduodenal ESD among the four DOACs, with rivaroxaban following. The low risk associated with dabigatran in gastric ESD aligns with findings from a prior nationwide multicenter study [21]. However, that earlier study did not evaluate the risk of delayed bleeding for other DOACs because of a limited number of cases nor did it examine dosage variation for each DOAC, even though dosage had minimal impact on the differences in delayed bleeding rates among the four DOACs. The different targets, i.e., thrombin for dabigatran and factor Xa for the three other DOACs, may have affected the varying rates of delayed bleeding among these medications. The risk of delayed bleeding in esophageal ESD followed a trend similar to that in gastroduodenal ESD. However, the small sample size likely contributed to the lack of significant results. A larger sample would be required to draw firm conclusions regarding esophageal ESD. Nonetheless, the trend in delayed bleeding risk among the four DOACs may remain consistent in upper GI ESD, given the relatively similar ORs between gastroduodenal and esophageal ESD.

A recent study on colorectal ESD in patients taking DOACs showed different findings [38], indicating that dabigatran had the highest risk of delayed bleeding in this context. Dabigatran’s behavior as a prodrug and the differences in acidic conditions between patients with gastric and colorectal ESD may explain these conflicting results. Dabigatran etexilate is more effectively absorbed in an acidic milieu in the GI tract. While it is formulated together with tartaric acid to reduce the variability in absorption, acidic conditions in the stomach can still impact its absorption. Indeed, two studies have shown that such conditions can significantly reduce the trough and peak plasma levels of dabigatran [39, 40]. Many cases with early gastric cancer have a nonacidic condition in the stomach due to gastric atrophy, which could reduce the absorption of dabigatran etexilate, resulting in a lower delayed bleeding rate and higher ischemic stroke rate after gastric ESD. Although there is no data on the gastric acidity in patients with colorectal ESD, differences in the acidity may contribute to the contradictory findings between gastric and colorectal ESD.

Second, and more importantly, we first revealed a lower risk of ischemic stroke with rivaroxaban and apixaban compared with dabigatran in upper GI ESD, especially when the DOACs were limited to the standard dose. A previous multicenter study involving over 10,000 cases with gastric ESD revealed a thromboembolic event rate of 0.03% with all events classified as ischemic stroke [13]. Further, all events occurred in patients on anticoagulants, regardless of the anticoagulant status, leading to an incidence rate of 0.52%. Thus, our study results are crucial, considering the risk of thromboembolic events in upper GI ESD. Although no studies have directly compared the risk of ischemic stroke among DOACs in patients with upper GI ESD, several studies have compared its risk in those with atrial fibrillation [18–20]. However, the results in the risk of ischemic stroke for each DOAC were inconsistent across the previous studies. Our results were also inconsistent with these reports. However, the different targets in the coagulation pathway between dabigatran and three other drugs, i.e., thrombin and factor Xa, respectively, may potentially explain the higher risk of ischemic stroke in dabigatran in this study.

Regarding an optimal DOAC for upper GI ESD, dabigatran exhibited the lowest risk of delayed bleeding in gastroduodenal ESD and also showed the lowest rate of delayed bleeding in esophageal ESD, although this finding was not statistically significant. However, it presented the highest risk of ischemic stroke in upper GI ESD, especially at standard doses. The guidelines [22] indicate that the risk of thromboembolism draws more attention than the risk of bleeding due to its seriousness. Thus, the risk of ischemic stroke should be prioritized over that of delayed bleeding, making dabigatran less preferable for use in upper GI ESD. Rivaroxaban, conversely, demonstrated a low risk of ischemic stroke in upper GI ESD, a second-lowest risk of delayed bleeding in gastroduodenal ESD, and a lower trend in risk for delayed bleeding in esophageal ESD. Altogether, rivaroxaban might be optimal for upper GI ESD. Although this study does not explain the reasons for the favorable results of rivaroxaban compared with the three other DOACs, especially apixaban and edoxaban, these results may help clinicians select a DOAC for upper GI ESD. In clinical practice, gastroenterologists typically do not prescribe DOACs but can recommend switching to rivaroxaban during the perioperative period for prescribing physicians, making our results relevant to clinical practice.

The strength of this study lies in the large number of cases, which enabled us to clarify the risk of ischemic stroke in each DOAC for the first time for upper GI ESD. Furthermore, the use of IPW and a generalized boosted model resulted in the best balance of baseline characteristics, including the risk of ischemic stroke and bleeding, among the four DOAC groups, ensuring reliable results.

This study had several limitations. First, this is not a randomized controlled trial but is a retrospective study, with the potential for selection bias. Second, the perioperative management strategy of DOACs (i.e., continuation, interruption, and heparin bridging) varied among patients. While heparin use was included as a covariate for IPW, this analysis did not consider DOAC continuation or interruption. Furthermore, the timing of interruption and resumption is important. Although the guidelines recommended stopping DOACs on the morning of ESD and resuming them the following morning [41], it is unclear how many cases complied with this recommendation. Third, some lesion-based factors, such as tumor size and location, which were reported as risk factors for delayed bleeding in gastric ESD [14], were not considered as covariates. Fourth, this study faced potential inaccuracies in coding including ischemic stroke, although a previous validation study demonstrated that the reliability of this database was relatively high in general [29] and the rate of ischemic stroke in patients on DOACs was similar to those in previous reports [13, 33]. Furthermore, the severity of ischemic stroke could not be assessed in this study, despite that it varies among the cases. Fifth, the results in cases that did not use heparin may best reflect the actual situation in the current clinical practice because the use of heparin was less recommended in the guidelines published in 2017 (in Japanese) [41], although this analysis was set as one of the sensitivity analyses in the present study. Indeed, only 8.9% of the cases used heparin after 2018. Nevertheless, the results in cases limited to no use of heparin were relatively consistent with the main analysis. Lastly, the results in some sensitivity analyses were not consistent with the main results, although the advantage of rivaroxaban was consistently observed in both the main and sensitivity analyses.

In conclusion, this large-scale database study revealed that rivaroxaban had the second lowest risk of delayed bleeding after dabigatran in gastroduodenal ESD, with relatively similar results observed in esophageal ESD. Additionally, rivaroxaban presented a lower risk of ischemic stroke than dabigatran in upper GI ESD, especially when limited to the standard dose. Given the greater importance of the risk of ischemic stroke over that of delayed bleeding, rivaroxaban might be optimal among the four DOACs for upper GI ESD.

## Supplementary Information

Below is the link to the electronic supplementary material.

Supplementary file1 (DOCX 39 KB)

## Abbreviations

ESD: Endoscopic submucosal dissection
GI: Gastrointestinal
DOACs: Direct oral anticoagulants
DPC: Diagnosis procedure combination
BMI: Body mass index
CCI: Charlson comorbidity index
PPIs: Proton pump inhibitors
P2Y12RA: P2Y12 receptor antagonist
NSAIDs: Nonsteroidal anti-inflammatory drugs
IPW: Inverse probability weighting
SMDs: Standardized mean differences
OR: Odds ratio
SD: Standard deviation
CI: Confidence interval
