# Supplementary material for: Optimal direct oral anticoagulant for upper gastrointestinal endoscopic submucosal dissection
Source: J Gastroenterol. 2024 Nov 27;60(1):66–76. doi: 10.1007/s00535-024-02171-2 (PMC11717785; doi:10.1007/s00535-024-02171-2)
Supplement: Supplementary file 1 — Supplementary file1 (DOCX 39 KB) [file 535_2024_2171_MOESM1_ESM.docx]

**Supplementary Table 1.** The characteristics and dosage reduction criteria of the four DOACs.

|  | **Dabigatran** | **Rivaroxaban** | **Apixaban** | **Edoxaban** |
| --- | --- | --- | --- | --- |
| Target | Thrombin | Factor Xa | Factor Xa | Factor Xa |
| Administration | Twice daily | Once daily | Twice daily | Once daily |
| Standard dose | 300 mg/day | 15 mg/day | 10 mg/day | 60 mg/day |
| Prodrug | Yes | No | No | No |
| Half-life | 14–17 hrs | 9–13 hrs | 8–15 hrs | 6–11 hrs |
| Reduction criteria | Age ≥ 70 years; CrCl 30–50 mL/min; concomitant use of P–glycoprotein inhibitors; history of GI bleeding | CrCl 15–49 mL/min | Patients with ≥ two of the following characteristics: Age ≥ 80 years; serum creatinine ≥ 1.5 mg/dL; body weight ≤ 60 kg | CrCl 15–50 mL/min; concomitant use of P–glycoprotein inhibitors; body weight ≤ 60 kg |
| Avoid | CrCl < 30 mL/min | CrCl < 15 mL/min | CrCl < 15 mL/min | CrCl < 15 mL/min |

DOAC, direct oral anticoagulant; CrCl, creatinine clearance; GI, gastrointestinal.

**Supplementary Table 2.** Participant characteristics according to the DOACs at standard dose.

|  |  |  |  |  | **SMD** | |
| --- | --- | --- | --- | --- | --- | --- |
|  | **Dabigatran** | **Rivaroxaban** | **Apixaban** | **Edoxaban** | **Before IPW** | **After IPW** |
| **Upper GI ESD** | ***n* = 251** | ***n* = 1,507** | ***n* = 1,312** | ***n* = 972** |  |  |
| Age (y), mean (SD) | 71.7 (7.24) | 73.5 (6.6) | 74.7 (6.09) | 74.9 (7.5) | 0.259 | 0.031 |
| Sex, *n* (%) |  |  |  |  | 0.056 | 0.021 |
| Male | 201 (91.8) | 1,100 (89.1) | 925 (88.6) | 700 (89.5) |  |  |
| Female | 50 (8.2) | 407 (10.9) | 387 (11.4) | 272 (10.5) |  |  |
| BMI (kg/m^2^), mean (SD) | 24.4 (3.5) | 24.1 (3.4) | 24.2 (4.5) | 24.3 (3.5) | 0.042 | 0.021 |
| CCI, mean (SD) | 0.9 (1.1) | 1.0 (1.1) | 1.1 (1.2) | 1.0 (1.3) | 0.086 | 0.018 |
| Hospital volume, mean (SD) | 73.0 (60.7) | 69.27 (55.8) | 69.1 (57.7) | 61.0 (49.4) | 0.109 | 0.025 |
| Drug use, *n* (%) |  |  |  |  |  |  |
| Heparin | 55 (21.9) | 218 (14.5) | 222 (16.9) | 150 (15.4) | 0.104 | 0.024 |
| Aspirin | 21 (8.4) | 125 (8.3) | 112 (8.5) | 101 (10.4) | 0.037 | 0.025 |
| P2Y12RA | 4 (1.6) | 54 (3.6) | 61 (4.6) | 29 (3.0) | 0.095 | 0.102 |
| Cilostazol | 3 (1.2) | 27 (1.8) | 30 (2.3) | 27 (2.8) | 0.063 | 0.021 |
| Other Antiplatelet drugs | 8 (3.2) | 44 (2.9) | 43 (3.3) | 29 (3.0) | 0.012 | 0.013 |
| PPIs | 223 (88.8) | 1,291 (85.7) | 1,118 (85.2) | 812 (83.5) | 0.079 | 0.024 |
| Vonoprazan | 112 (44.6) | 784 (52.0) | 731 (55.7) | 589 (60.6) | 0.174 | 0.034 |
| Mucosal protective agents | 167 (66.5) | 1,021 (67.8) | 912 (69.5) | 667 (68.6) | 0.035 | 0.019 |
| NSAIDs | 26 (10.4) | 103 (6.8) | 95 (7.2) | 54 (5.6) | 0.092 | 0.043 |
| Corticosteroids | 20 (8.0) | 89 (5.9) | 77 (5.9) | 62 (6.4) | 0.045 | 0.028 |
| CHADS2 score | 1.1 (1.1) | 1.2 (1.0) | 1.4 (1.1) | 1.3 (1.1) | 0.147 | 0.067 |
| **Gastroduodenal ESD** | ***n* = 204** | ***n* = 1,200** | ***n* = 1,023** | ***n* = 777** |  |  |
| Age (y), mean (SD) | 72.1 (7.4) | 74.0 (6.5) | 75.1 (6.1) | 75.5 (7.5) | 0.274 | 0.038 |
| Sex, *n* (%) |  |  |  |  | 0.058 | 0.048 |
| Male | 187 (91.7) | 1,068 (89.0) | 904 (88.4) | 695 (89.4) |  |  |
| Female | 17 (8.3) | 132 (11.0) | 119 (11.6) | 82 (10.6) |  |  |
| BMI (kg/m^2^), mean (SD) | 24.4 (3.6) | 24.2 (3.3) | 24.6 (4.7) | 24.5 (3.6) | 0.048 | 0.050 |
| CCI, mean (SD) | 0.9 (1.1) | 0.9 (1.1) | 1.1 (1.2) | 1.0 (1.2) | 0.092 | 0.013 |
| Hospital volume, mean (SD) | 81.6 (62.1) | 78.4 (56.9) | 79.3 (59.7) | 69.1 (50.3) | 0.114 | 0.049 |
| Drug use, *n* (%) |  |  |  |  |  |  |
| Heparin | 42 (20.6) | 168 (14.0) | 170 (16.6) | 117 (15.1) | 0.095 | 0.034 |
| Aspirin | 15 (7.4) | 103 (8.6) | 90 (8.8) | 84 (10.8) | 0.062 | 0.043 |
| P2Y12RA | 2 (1.0) | 46 (3.8) | 47 (4.6) | 22 (2.8) | 0.122 | 0.123 |
| Cilostazol | 3 (1.5) | 24 (2.0) | 27 (2.6) | 18 (2.3) | 0.045 | 0.015 |
| Other Antiplatelet drugs | 7 (3.4) | 32 (2.7) | 32 (3.1) | 26 (3.3) | 0.024 | 0.029 |
| PPIs | 185 (90.7) | 1030 (85.8) | 876 (85.6) | 650 (83.7) | 0.107 | 0.055 |
| Vonoprazan | 98 (48.0) | 676 (56.3) | 634 (62.0) | 500 (64.4) | 0.185 | 0.044 |
| Mucosal protective agents | 139 (68.1) | 823 (68.6) | 742 (72.5) | 543 (69.9) | 0.053 | 0.021 |
| NSAIDs | 15 (7.4) | 64 (5.3) | 60 (5.9) | 40 (5.1) | 0.049 | 0.027 |
| Corticosteroids | 11 (5.4) | 49 (4.1) | 45 (4.4) | 41 (5.3) | 0.038 | 0.009 |
| CHADS2 score | 1.2 (1.0) | 1.3 (1.0) | 1.5 (1.1) | 1.4 (1.1) | 0.155 | 0.064 |

DOACs, direct oral anticoagulants; SMD, standardized mean difference; IPW, inverse probability weighting; ESD, endoscopic submucosal dissection; SD, standard deviation; BMI, body mass index; CCI, Charlson comorbidity index; P2Y12RA, P2Y12 receptor antagonist; PPIs, proton pump inhibitors; NSAIDs, nonsteroidal anti-inflammatory drugs.

**Supplementary Table 3.** Participant characteristics according to the DOACs when participants were limited to no use of heparin.

|  |  |  |  |  | **SMD** | |
| --- | --- | --- | --- | --- | --- | --- |
|  | **Dabigatran** | **Rivaroxaban** | **Apixaban** | **Edoxaban** | **Before IPW** | **After IPW** |
| **Upper GI ESD** | ***n* = 872** | ***n* = 2,600** | ***n* = 2,494** | ***n* = 2,200** |  |  |
| Age (y), mean (SD) | 75.8 (6.8) | 76.2 (7.0) | 77.7 (6.8) | 77.0 (7.3) | 0.158 | 0.045 |
| Sex, *n* (%) |  |  |  |  | 0.145 | 0.039 |
| Male | 785 (90.0) | 2,213 (85.1) | 2,083 (83.5) | 1,767 (80.3) |  |  |
| Female | 87 (10.0) | 387 (14.9) | 411 (16.5) | 433 (19.7) |  |  |
| BMI (kg/m^2^), mean (SD) | 23.7 (3.4) | 23.7 (3.43) | 23.5 (4.1) | 23.3 (3.6) | 0.062 | 0.027 |
| CCI, mean (SD) | 0.9 (1.0) | 1.0 (1.1) | 1.1 (1.2) | 1.1 (1.2) | 0.088 | 0.048 |
| Hospital volume, mean (SD) | 73.4 (58.7) | 68.0 (55.3) | 67.8 (58.2) | 65.4 (51.6) | 0.072 | 0.007 |
| Drug use, *n* (%) |  |  |  |  |  |  |
| Aspirin | 70 (8.0) | 259 (10.0) | 246 (9.9) | 215 (9.8) | 0.034 | 0.012 |
| P2Y12RA | 30 (3.4) | 97 (3.7) | 123 (4.9) | 64 (2.9) | 0.055 | 0.024 |
| Cilostazol | 12 (1.4) | 47 (1.8) | 60 (2.4) | 55 (2.5) | 0.048 | 0.038 |
| Other Antiplatelet drugs | 32 (3.7) | 72 (2.8) | 74 (3.0) | 67 (3.0) | 0.026 | 0.010 |
| PPIs | 761 (87.3) | 2,198 (84.5) | 2,081 (83.4) | 1,796 (81.6) | 0.083 | 0.009 |
| Vonoprazan | 392 (45.0) | 1,367 (52.6) | 1,413 (56.7) | 1,380 (62.7) | 0.194 | 0.033 |
| Mucosal protective agents | 596 (68.3) | 1,778 (68.4) | 1,700 (68.2) | 1,474 (67.0) | 0.015 | 0.013 |
| NSAIDs | 54 (6.2) | 166 (6.4) | 155 (6.2) | 149 (6.8) | 0.013 | 0.012 |
| Corticosteroids | 45 (5.2) | 141 (5.4) | 144 (5.8) | 177 (8.0) | 0.061 | 0.016 |
| CHADS2 score | 1.4 (1.1) | 1.4 (1.0) | 1.6 (1.1) | 1.5 (1.1) | 0.105 | 0.041 |
| **Gastroduodenal ESD** | ***n* = 745** | ***n* = 2,128** | ***n* = 2,033** | ***n* = 1,775** |  |  |
| Age (y), mean (SD) | 76.3 (6.7) | 76.7 (6.9) | 78.2 (6.7) | 77.7 (7.1) | 0.165 | 0.049 |
| Sex, *n* (%) |  |  |  |  | 0.154 | 0.051 |
| Male | 663 (89.0) | 1,771 (83.2) | 1,661 (81.7) | 1,388 (78.2) |  |  |
| Female | 82 (11.0) | 357 (16.8) | 372 (18.3) | 387 (21.8) |  |  |
| BMI (kg/m^2^), mean (SD) | 23.8 (3.4) | 23.8 (3.4) | 23.7 (4.2) | 23.6 (3.6) | 0.042 | 0.029 |
| CCI, mean (SD) | 0.9 (1.0) | 1.0 (1.1) | 1.1 (1.2) | 1.0 (1.2) | 0.103 | 0.049 |
| Hospital volume, mean (SD) | 80.9 (59.7) | 75.9 (56.6) | 76.0 (60.2) | 73.9 (52.3) | 0.061 | 0.006 |
| Drug use, *n* (%) |  |  |  |  |  |  |
| Aspirin | 60 (8.1) | 226 (10.6) | 206 (10.1) | 183 (10.3) | 0.045 | 0.024 |
| P2Y12RA | 25 (3.4) | 81 (3.8) | 107 (5.3) | 48 (2.7) | 0.070 | 0.022 |
| Cilostazol | 12 (1.6) | 42 (2.0) | 53 (2.6) | 45 (2.5) | 0.041 | 0.037 |
| Other Antiplatelet drugs | 29 (3.9) | 55 (2.6) | 60 (3.0) | 55 (3.1) | 0.038 | 0.003 |
| PPIs | 653 (87.7) | 1,800 (84.6) | 1,698 (83.5) | 1,450 (81.7) | 0.088 | 0.021 |
| Vonoprazan | 351 (47.1) | 1,198 (56.3) | 1,250 (61.5) | 1,189 (67.0) | 0.221 | 0.040 |
| Mucosal protective agents | 518 (69.5) | 1,466 (68.9) | 1,420 (69.8) | 1,214 (68.4) | 0.018 | 0.014 |
| NSAIDs | 38 (5.1) | 114 (5.4) | 111 (5.5) | 111 (6.3) | 0.026 | 0.025 |
| Corticosteroids | 31 (4.2) | 92 (4.3) | 102 (5.0) | 122 (6.9) | 0.065 | 0.032 |
| CHADS2 score | 1.4 (1.0) | 1.4 (1.0) | 1.6 (1.1) | 1.5 (1.1) | 0.117 | 0.038 |
| **Esophageal ESD** | ***n* = 127** | ***n* = 472** | ***n* = 461** | ***n* = 425** |  |  |
| Age (y), mean (SD) | 72.7 (6.8) | 74.2 (7.2) | 75.4 (6.8) | 74.3 (7.4) | 0.201 | 0.040 |
| Sex, *n* (%) |  |  |  |  | 0.147 | 0.093 |
| Male | 122 (96.1) | 442 (93.6) | 422 (91.5) | 379 (89.2) |  |  |
| Female | 5 (3.9) | 30 (6.4) | 39 (8.5) | 46 (10.8) |  |  |
| BMI (kg/m^2^), mean (SD) | 22.9 (3.2) | 23.2 (3.3) | 22.8 (3.3) | 22.3 (3.2) | 0.144 | 0.048 |
| CCI, mean (SD) | 1.1 (1.2) | 1.0 (1.2) | 1.1 (1.2) | 1.2 (1.4) | 0.074 | 0.042 |
| Hospital volume, mean (SD) | 29.6 (22.9) | 32.3 (29.1) | 31.7 (27.7) | 30.2 (28.3) | 0.059 | 0.049 |
| Drug use, *n* (%) |  |  |  |  |  |  |
| Aspirin | 10 (7.9) | 33 (7.0) | 40 (8.7) | 32 (7.5) | 0.034 | 0.029 |
| P2Y12RA | 5 (3.9) | 16 (3.4) | 16 (3.5) | 16 (3.8) | 0.017 | 0.029 |
| Cilostazol | 0 (0.0) | 5 (1.1) | 7 (1.5) | 10 (2.4) | 0.124 | 0.110 |
| Other Antiplatelet drugs | 3 (2.4) | 17 (3.6) | 14 (3.0) | 12 (2.8) | 0.039 | 0.017 |
| PPIs | 108 (85.0) | 398 (84.3) | 383 (83.1) | 346 (81.4) | 0.054 | 0.006 |
| Vonoprazan | 41 (32.3) | 169 (35.8) | 163 (35.4) | 191 (44.9) | 0.132 | 0.058 |
| Mucosal protective agents | 78 (61.4) | 312 (66.1) | 280 (60.7) | 260 (61.2) | 0.057 | 0.052 |
| NSAIDs | 16 (12.6) | 52 (11.0) | 44 (9.5) | 38 (8.9) | 0.067 | 0.042 |
| Corticosteroids | 14 (11.0) | 49 (10.4) | 42 (9.1) | 55 (12.9) | 0.065 | 0.054 |
| CHADS2 score | 1.2 (1.1) | 1.1 (1.0) | 1.3 (1.1) | 1.3 (1.1) | 0.101 | 0.083 |

DOACs, direct oral anticoagulants; SMD, standardized mean difference; IPW, inverse probability weighting; ESD, endoscopic submucosal dissection; SD, standard deviation; BMI, body mass index; CCI, Charlson comorbidity index; P2Y12RA, P2Y12 receptor antagonist; PPIs, proton pump inhibitors; NSAIDs, nonsteroidal anti-inflammatory drugs.
